# Tumor suppressor gene methylation in follicular lymphoma: a comprehensive review

**DOI:** 10.1186/1476-4598-5-44

**Published:** 2006-10-06

**Authors:** John Hayslip, Alberto Montero

**Affiliations:** 1 Hollings Cancer Center, Medical University of South Carolina, Clinical Sciences Building Room 903, PO Box 250635, Charleston, SC 29425, USA

## Abstract

Transcriptional silencing of tumor suppressor genes, associated with DNA methylation, is a common epigenetic event in hematologic malignancies. Although DNA hypermethylation of CpG islands is well described in acute leukemias and myelodysplastic syndromes, much less is known of the specific methylation changes that commonly occur in follicular B cell lymphomas. Earlier methylation studies of follicular lymphoma involved only cell lines; however there is a growing literature of methylation changes in primary human FL samples. Published studies of primary follicular lymphoma specimens have demonstrated that: androgen receptor, SHP1, and death-associated protein kinase genes are commonly methylated. By contrast, the cyclin dependent kinase inhibitors p15, p16, and p57 are uncommon epigenetic events in follicular lymphoma. Methylation of cyclin dependent kinase inhibitors is more common in high grade lymphomas, and may be an important step in the progression and transformation of follicular lymphoma. Further methylation studies in follicular lymphoma should investigate the prognostic and therapeutic significance of these epigenetic changes and investigate methylation of other genes. Finally, reactivation of methylated tumor suppressor genes through the use of hypomethylating agents is a promising and novel approach to the treatment of indolent and transformed follicular lymphomas.

## Background

Follicular lymphoma (FL), an indolent subgroup of non-hodgkin lymphomas, is a monoclonal lymphoid neoplasm arising from a malignant germinal center B lymphocyte. FL has an average annual incidence rate of 2.6 per 100,000 people and a median survival of 7.8 years [1,2].

Recent molecular studies have established that activation of various oncogenes and silencing of tumor suppressor genes is required for FL development and progression. Here we discuss published literature regarding transcriptional silencing associated with DNA methylation in FL. In particular, we detail the genes known to be frequently methylated in FL, discuss their associated protein's function, and conclude with considerations for incorporating hypomethylating agents to reactivate methylated tumor suppressor genes as a novel therapeutic strategy for indolent and transformed FL.

### Cancer genetics and epigenetics

#### Tumor suppressor genes

Tumor suppressor genes protect cells from undergoing malignant transformation. Tumor suppressor genes function by one of the following mechanisms: protect the genome from mutagenic events, impede dysregulated progression through the cell cycle, induce apoptosis in cells that escape normal cell cycle controls, and inhibit cellular migration and metastasis. Classically, tumor suppressor genes have been described to acquire loss of function mutations or deletions leading to their inability to impede malignant transformation. Alternatively, epigenetic events, such as methylation, represent a distinct mechanism of tumor suppressor gene inactivation. Aberrant gene promoter methylation is associated with gene silencing and is functionally equivalent to a deleted gene. Gene silencing by DNA methylation has been considered to be permanent in non-embryonic cells, only reversible pharmacologically during cell division. Interestingly, new findings in lymphocytes may challenge this paradigm of irreversibility but have yet to be widely replicated nor specifically studied in FL [3].

#### Promoter methylation down regulates transcription

The promoter region of a gene is located upstream, at the 5' end, from the transcription start site. Promoter regions of genes are more rich in cytosine-guanine dinucleotides than would be expected by chance alone, relative to the rest of the genome [4]. These cytosine guanine rich regions are referred to as CpG islands. Many mammalian genes have CpG islands in their promoter regions and are not methylated if the gene is to be transcribed within the cell. Methylation of cytosine residues in CpG islands, by DNA methyltransferase enzymes, is associated with transcriptional silencing.

Tumor suppressor gene methylation is a well recognized mechanism of oncogenesis in many tumor types. B-cell malignancies seem to be particularly susceptible to this phenomenon [5]. Although much of what is known about gene methylation in lymphoma has been derived from study of tumor cell lines, an increasing number of published studies have recently investigated primary human tumor samples. We review the current published literature of aberrant tumor suppressor gene methylation in primary human FL.

#### DNA methylation analysis techniques

Several methods to evaluate cytosine methylation are available and a complete review of these techniques is beyond the scope of this article. However, a succinct and thorough review of these methods is available [6]. The most commonly utilized methodologies for distinguishing methylated from unmethylated DNA all rely on DNA treatment with sodium bisulfite, which results in the conversion of all unmethylated cytosines to uracil, while leaving methylated cytosines unaltered. Two commonly utilized methods for quantification of DNA methylation are methylation specific polymerase chain reaction (MSP) and restriction enzyme-related polymerase chain reaction (ERP). Both methods require polymerase chain reactions (PCR) amplification of a specific locus within the CpG island of the gene of interest. MSP is a rapid and very sensitive technique to screen methylation where PCR primers designed to amplify either methylated or unmethylated bisulfite converted DNA are utilized; the intensities of methylated and unmethylated bands are analyzed by gel electrophoresis. The primary advantage of MSP is that it is thus far the most sensitive method and can detect 0.1% methylation. The disadvantages of this approach include: PCR bias (differential ability of methylated or unmethylated PCR primers to amplify gene product) and semi-quantitative nature. ERP utilizes methylation specific endonucleases to differentially cleave DNA fragments prior to undergoing PCR. ERP is not a widely used methodology due to problems with sensitivity.

### Commonly methylated genes in FL

#### Androgen receptor

The androgen receptor gene, located on the X chromosome, encodes a member of the receptor group that binds and mediates the actions of androgens [7]. When bound and activated by an androgen, the androgen receptor enters the nucleus and acts as a ligand-dependent transcription factor to upregulate dependent gene transcription [7]. This pathway appears important in regulating lymphopoiesis. Thymus size normally decreases during puberty as a result of increased levels of sex hormones. By contrast, in castrated males thymic hypertrophy has been reported [8]. Whether from decreased androgen production or loss of receptor function, loss of the androgen signal leads to increased release of immature B cells from the bone marrow into the peripheral blood [9].

DNA promoter hypermethylation of the androgen receptor gene is a common finding in FL and other lymphomas. McDonald and colleagues reported 16/19 samples of T and B lymphomas positive for methylated androgen receptor genes [10]. A similar analysis of grade I and II FL found 25/26 samples tested were positive for methylation at the *androgen receptor *promoter region [11]. This consistent finding suggests that *androgen receptor *promoter methylation may be a common epigenetic phenomenon in FL pathogenesis.

#### SHP1

SHP1, also known as PTP1C, PTPN6, HCP, and SHPTP1, is a phosphotyrosine phosphatase that plays many important roles in regulating immune system cell differentiation and activation [12,13]. SHP1 acts as a growth inhibitor in B-cells by down regulating the intracellular effects of immunoglobulin binding thus requiring more receptor binding to initiate B-cell activation and proliferation [12,13]. Conversely, B lymphocytes with decreased SHP1 activity are more likely to proliferate and escape apoptosis [12,13]. Methylation of *SHP1 *promoter region appears common across a variety of lymphomas. Most FL samples, 32 of 33 assayed, were methylated at the *SHP1 *promoter region [14,15]. Additionally, *SHP1 *was methylated in 100% of plasmacytomas, 93 % of diffuse large B cell lymphomas, 82% of MALT lymphomas, and 82% of mantle cell lymphomas [14,15]. Conversely, no promoter methylation of *SHP1 *was noted in 20 normal reactive lymph node samples [14]. Both the nearly uniform methylation of *SHP1 *in FL samples and the high rates across several lymphoma types suggest that down regulation of *SHP1 *by promoter hypermethylation may be an important step in lymphomagenesis.

#### DAP kinase

Death-associated protein kinase (DAPK) is a calcium-calmodulin-dependent serine/threonine kinase that participates in apoptosis [16]. DAPK was first demonstrated to participate in interferon-γ induced apoptosis [17]. Subsequently, DAPK has also been shown to contribute to tumor necrosis factor-α and Fas-induced apoptosis [18]. Blocking *DAPK *transcription in cells protects them from interferon-γ induced apoptosis but not the cell cycle inhibitory effects of interferon-γ [16]. Loss of *DAPK *expression due to gene hypermethylation has been demonstrated in bladder and renal cell carcinoma cell lines, immortalized B-cell lines, and primary, non-immortalized, B-cell lymphoma samples [19,20].

Methylation of *DAPK *may be a common epigenetic event in FL. In two published studies, twenty-five of twenty-nine (86%) FL samples were positive for aberrant *DAPK *methylation [5,21]. DAPK protein was not expressed in those lymphomas with *DAPK *methylation [5]. Similarly, in nine samples of transformed FL studied before and after transformation, 7/9 were found to have aberrant methylation of *DAPK *both before and after transformation [5]. Neither of the two unmethylated samples became methylated during the transformation [5]. These findings are consistent with the hypothesis that methylation of *DAPK *is a common, early epigenetic phenomenon of FL that allows these cells to escape the normal apoptosis of a non-antigen stimulated expanded b-cell population. However, there is also a correlation between increased *DAPK *methylation and increasing age as a potential confounding variable in such studies.

### Methylated cyclin dependent kinases inhibitors associated with FL transformation

#### p16

*p16*, located on chromosome 9p21, is the most commonly altered gene in human malignancies [22]. *p16 *is also known by the following names: *INK4*, *INK4A*, *CDK4I*, *MTS1*, and *CDKN2 *[22]. p16 is a 148 amino acid protein containing 4 ankyrin repeats [23]. p16 inhibits the phosphorylation of retinoblastoma protein and thereby impedes mitosis at G_1_-S transition of the cell cycle pathway [23]. By blocking unregulated cellular proliferation, p16 functions as an important tumor suppressor. The loss of p16 activity, either through gene mutation or promoter hypermethylation, is a common step in tumor development and progression.

Although homozygous deletion of *p16 *is common in numerous malignancies, it is uncommon in patients with newly diagnosed FL. Only two published studies have tested for *p16 *deletions in FL. Of twenty-nine FL cases tested, none showed the deletion [24,25]. However, one of nine patients with transformed FL did have a homozygous *p16 *deletion [25]. Little data exists examining *p16 *hypermethylation in FL. A combined analysis of published data showed aberrant *p16 *promoter methylation in five of sixteen FL cases [26,27]. Although not specifically reported as FL, another report of patients with "NHL-low grade" found one of eleven patients had tumors with detectible *p16 *promoter methylation [28].

However, *p16 *hypermethylation appears to be more frequent in lymphomas with a higher growth fraction. In two studies, fourteen of twenty-four high grade lymphoma samples were demonstrated *p16 *hypermethylation [27,28]. These findings suggest that *p16 *methylation may be an important epigenetic event in the progression and transformation of FL [25-27].

#### p15

*p15*, also known as *INK4B*, *MTS2 *and *CDKN2B*, is located 25 kilobases from the *p16 *gene and its protein shares significant areas of homologous amino acid sequences with p16 [29]. p15 inhibits cyclin dependent kinases 4 and 6 contributing to cell cycle arrest at the G_1_-S transition [30]. In contrast to *p16*, *p15 *expression is induced when cells are exposed to transforming growth factor-β (TGF-β) and may be essential to regulating the inhibitory cell cycle effects of this cytokine [29]. Similarly, the loss of *p15 *expression may contribute to TGF-β independent, dysregulated lymphopoeisis. However, presently it is unclear whether *p15 *is an independent tumor suppressor gene, as most studies of primary tumor samples reporting *p1*5 deletion have found simultaneous deletion of *p16 *also [31-33].

Methylation of the *p15 *promoter region has been reported in acute myeloid and lymphoblastic leukemias, multiple myeloma, and lymphomas [27,28,34,35]. Few studies have specifically detailed the histologic subtype of the lymphomas analyzed. Of those that have, ten of twenty-seven FL samples were found to have detectable *p15 *methylation [27,35]. Of the *p15 *methylated samples, 6/10 were also found to have *p16 *methylation [27,35]. The high concordance of *p15 *and *p16 *methylation makes it difficult to determine the independent importance of either gene alone.

#### p57

p57, also known as KIP2 or CDKN1C, is a cyclin-dependant kinase inhibitor. The *p57 *gene is located on chromosome 11p15.5 [36]. p57 is a tight-binding inhibitor of several G_1 _cyclin/Cdk complexes and a negative regulator of cell proliferation. Mutations of *p57 *are implicated in sporadic cancers and Beckwith-Wiedemann syndrome suggesting that it is a tumor suppressor gene [37-39]. In a recent study, eight of eighteen FL samples (44%) had methylation of the *p57 *promoter region [40]. A similar proportion of *p57 *methylation is found in diffuse large B-cell lymphoma samples [40].

#### p14 is not commonly methylated

p14 protein, also known as p19^ARF ^or CDKN2A, is also a cyclin-dependent kinase inhibitor known to induce cell cycle arrest at G_1 _and G_2 _[41]. p14 is encoded by three exons designated 1-β, 2, and 3 [41]. Exon 2 and 3 is shared with p16 although exon 1 is unique for each of the proteins [41]. Mice with homozygous deletion of *p14 *frequently develop numerous tumors including lymphomas [42]. Despite these interesting findings, Baur and colleagues found no methylation of the *p14 *promoter region 5' to exon 1-β in 56 lymphoma samples, which included 14 FL samples [27]. Therefore, hypermethylation of *p14 *does not appear to be a common epigenetic event in FL.

### Methylation of genes with developing interest

#### GSTP1

GSTP1 is an important enzyme in the detoxification of mutagens [43,44]. Different polymorphisms of *GSTP1 *have been associated with the development of lymphoma, suggesting that *GSTP1 *may be important in the development and/or progression of lymphoma in humans [45]. Less active polymorphisms of *GSTP1 *confer less resistance to lymphoma development. In one study evaluating promoter methylation of *GSTP1*, ten of eighteen FL samples were found to have *GSTP1 *methylation [5]. Similarly, *GSTP1 *methylation was detected in six of eight hairy cell leukemia samples; by contrast, no methylation was detected in twelve chronic lymphocytic leukemia samples [5]. Further study in FL is warranted as the down regulation of *GSTP1 *may be an early event in lymphomagenesis.

#### IL-12R β-2

Interleukin-12 is a pro-inflammatory cytokine produced by dendritic cells and phagocytes that promotes production of interferon-γ and acts synergistically with other cytokines to augment the proliferation and activity of T and NK cells [46]. The direct effects of IL-12 on B cells are not fully understood, but may act through the NFκB pathway in naïve B cells to produce interferon-γ [47]. The IL-12 receptor is composed of two subunits: β-1 and β-2 [48]. The β-1 subunit can also dimerize in the formation of IL-23 receptor, but the β-2 subunit is unique to the IL-12 receptor [46]. Although the β-2 subunit is normally expressed on the surface of B-lymphocytes, its expression has not been detected in various malignant B cell lines, suggesting its potential importance in malignant progression [49].

In a recent study, IL-12 receptor β-2 was not expressed in 12 FL samples tested [50]. Two of these samples were tested for DNA methylation of the IL-12 receptor β-2 gene, and both found to be methylated [50]. Results for mantle cell lymphoma, marginal zone lymphoma, and B cell chronic lymphocytic leukemia (CLL) were similarly methylated [50]. Incubation with 5-aza-2'-deoxycytidine, a demethylating agent, resulted in the re-expression of IL-12 receptor β-2 in FL and CLL cells [50]. Although the mechanism is not entirely clear, these data suggest IL-12 may serve as a tumor suppressor across a wide variety of B cell malignancies and methylation of IL-12 receptor β-2 gene may be a common step in the development of B cell malignancies.

#### Snk plk2

Polo-like kinase 2 (Snk/Plk2) is a serine-threonine kinase that contributes to cells transitioning from G_1 _to S phase [51]. The gene is located at 5q12.1-q13.2 [52]. Although cells lacking this enzyme are more sensitive to paclitaxel induced cell death, it is unclear how this enzyme acts as a tumor suppressor [52]. *Snk/Plk2 *promoter region was found to be methylated in two of four FL samples in one study [53]. The significance of these findings and the impact of epigenetic regulation of *Snk/Plk2 *on lymphomagenesis still need to be further explored.

#### GADD45-γ

GADD45 proteins α, β, and γ, are induced by DNA damage and extrinsic stressors [54]. GADD45-γ exerts at least part of its influence by inhibiting the Cdk1/cyclinB1 complex and blocking the S and G_2_-M cell cycle transition [55]. Tumor cell lines previously lacking GADD45-γ expression that were transfected with *GADD45-γ *showed significant reduction in colony formation and some cases with frank apoptosis and no culture growth [56]. Gene methylation of *GADD45-γ *has only rarely been reported in primary FL samples. In one recent study, although 11/13 non-hodgkin lymphoma cell lines were *GADD45-γ *methylated, only 1/6 primary FL samples were found to be methylated [56]. The apparent discordance between cell lines and primary patient samples will require further evaluation to better estimate the prevalence of *GADD45-γ *methylation in FL.

#### Annexin 1

Annexin 1 is a calcium and phospholipid binding protein that may participate in both spontaneous and stimulated apoptosis in some cell lines [57]. Although annexin 1 is expressed in normal adenoid B-cells, it is not detected in FL cell lines or burkitt lymphoma cell lines [58]. The *annexin 1 *gene was analyzed in burkitt lymphoma cell lines and found to be structurally intact but methylated [58]. Annexin 1 was re-expressed when these burkitt lymphoma cells were cultured with 5-aza-2'-deoxycytidine suggesting that aberrant *annexin 1 *methylation explains decreased annexin 1 expression in FL cells. [58]. Additional studies on patient FL samples are needed to better clarify whether *annexin 1 *methylation is a common epigenetic event in FL.

#### Treatment strategies

With combination chemotherapy and rituximab, FL patients may have long remissions. Unfortunately, relapses are part of the natural history of FL. With the exception of treatment with allogeneic bone marrow transplant or stage I disease that is irradiated or resected, FL is generally considered incurable. Most patients will experience multiple relapses of their lymphoma. Patients generally have a shorter response to chemotherapy with each episode of relapsing disease. Further, patients are at risk for their lymphoma transforming from an indolent FL to a more aggressive and treatment refractory lymphoma as the disease progresses.

#### Hypomethylating agents

Azacitidine (5-azacytidine) [59] and decitabine (5-aza-2'-deoxycytidine) [60] are two hypomethylating medications approved by the United States Food and Drug Administration for the treatment of myelodysplastic syndromes. Other similar compounds have been described but are not employed clinically in the United States [61]. At concentrations above those required to achieve demethylation, these cytidine analogues have direct cytotoxicity by inhibiting DNA synthesis [59,62]. However, clinically these agents are believed to be incorporated into replicating DNA and inhibit DNA methyltransferases (DNMT) [59,62]. During normal cellular replication, the methylation status of each gene is well conserved from the parent to daughter cells. When DNA methyltransferases are inhibited, the newly replicated DNA cannot be methylated by DNMT, allowing transcription and translation of the associated gene. Although beyond the scope of this article, a full discussion of the mechanism of action of this class of medications is available [63]. There is a theoretical concern that treatment with hypomethylating agents may induce the expression of silenced oncogenes or allow translocation of transposable elements within the genome [64]. However, in clinical trials of azacitidine in patients with myelodysplastic syndrome, progression to leukemia was reduced with treatment suggesting that if present, the mutagenic risk of hypomethylation is smaller than the therapeutic benefit in that clonal malignancy [65].

#### Strategies for therapeutic demethylation

We have reviewed the growing body of clinical data detailing genes known to be hypermethylated in FL. Although no clinical trial has specifically investigated hypomethylating agents in patients with FL, individual patients with various lymphomas have been included in early development trials of these agents [66-68]. Some partial responses were reported in these single agent trials, although no documented response lasted longer than seven months and the type of lymphomas are not well described [66-68]. In the future, four unique settings exist to investigate hypomethylating agents in FL: i. refractory disease; ii. transformed lymphoma; iii. alternative to traditional cytotoxic chemotherapy; iv. adjuvant to induction cytotoxic chemotherapy.

Patients with refractory cancers are often considered for early development trials as they have already failed standard therapies. Given lymphoma patients short duration of response in phase I trials of DNMT inhibitors, we expect DNMT inhibitors will be most active when incorporated into a multi-drug regimen. Additionally, FL transformation is associated with aberrant methylation of cyclin dependent kinase inhibitors and therapeutic reversal should be explored in this setting. Another appealing strategy is to treat FL patients without cytotoxic therapies. If the hypomethylating agents can induce the expression of androgen receptor, DAPK, and IL-12 receptor β-2, then concurrent treatment with their respective ligands may be investigated clinically. Finally, if a clinical benefit is demonstrated in these settings, DNMT inhibitors could be evaluated as an adjuvant to FL induction chemotherapy regimens.

## Conclusion

*Androgen receptor*, *SHP1*, and *DAPK *are commonly methylated genes in FL. We have described a growing list of additional genes that may be frequently methylated in FL and are under investigation. Additionally, methylation of cyclin dependent kinase inhibitors *p16*, *p15*, and *p57 *may be associated with transformation of FL to a more aggressive lymphoma in some patients. More research is needed to identify epigenetic events important to lymphomagenesis and progression as well as identifying prognostic relevance. Clinical trials to develop therapeutic strategies with hypomethylating agents are needed. Preclinical data with hypomethylating agents in FL are promising and offer a new paradigm for clinical exploration in the treatment of patients with FL.

**Table 1:** Methylated genes in primary follicular lymphoma samples

Gene	# of Methylated Samples (%)	Method of analysis	Gene function	Reference
Androgen receptor	25/26 (96)	MSP	Ligand-dependent transcription factor	[11]
SHP1	32/33 (97)	MSP	Inhibits intracellular effects of surface immunoglobulin binding in B lymphocytes	[14, 15]
Death-associated protein kinase	25/29 (86)	MSP	Involved in interferon-γ, tumor necrosis factor-α, and Fas-ligand induced apoptosis	[5, 21]
p16	5/16 (31)	ERP	Cyclin dependent kinase inhibitor	[26, 27]
p15	10/27 (37)	ERP	Cyclin dependent kinase inhibitor	[27, 35]
p57	8/18 (44)	MSP	Cyclin dependent kinase inhibitor	[40]
GSTP1	10/18 (56)	MSP	Detoxify mutagens	[5]
IL-12 receptor β-2	2/2 (100)	MSP	Forms heterodimer with IL-12Rβ1 to form IL-12 receptor	[50]
Snk/plk2	2/4 (50)	MSP	DNA damage response and cell growth arrest	[53]
GADD45-γ	1/6 (17)	MSP	DNA damage response and cell growth arrest	[56]

Abbreviations. MSP: Methylation specific PCR; ERP: Restriction enzyme-related PCR
